# Supplementary material for: Rapid, Portable, and Electricity-free Sample Extraction Method for Enhanced Molecular Diagnostics in Resource-Limited Settings
Source: Anal Chem. 2024 Jul 5;96(28):11181–8. doi: 10.1021/acs.analchem.4c00319 (PMC11256010; doi:10.1021/acs.analchem.4c00319)
Supplement: Supplementary file 1 — ac4c00319_si_001.pdf [file ac4c00319_si_001.pdf]

Supporting information

A Rapid, Portable, and Electricity-Free Sample Extraction Method for Enhanced Molecular Diagnostics in Resource-Limited Settings

Ivana Pennisi[a,b,#], Matthew L. Cavuto[a,b,#], Luca Miglietta[a], Kenny Malpartida-Cardenas[a], Oliver W Stringer[a], Katerina-Theresa Mantikas [b], Ruth Reid[a], Rebecca Frise[a], Nicolas Moser[b], Paul Randell[c], Frances Davies[c], Frances Bolt[a], Wendy Barclay[a], Alison Holmes[a], Pantelis Georgiou[b], Jesus Rodriguez-Manzano[a*]

[a]Department of Infectious Disease, Faculty of Medicine, Imperial College London, SW72AZ, UK
[b]Department of Electrical and Electronic Engineering, Faculty of Engineering, Imperial College, London, SW72BT, UK
[c]Imperial College Healthcare NHS Trust, Charing Cross Hospital, London, W6 8RP, UK
#These authors contributed equally to the work.

Table of contents

Supplementary tables.......2
Table S1. CDC RT-qPCR panel: primers and probes.......2
Table S2. Clinical sample characterization by CDC RT- qPCR, based on N1 CDC assay.......3
Supplementary figures.......4
Figure S1. SmartLid workflow.......4
Figure S2. RNA concentration in clinical samples.......5

Supplementary tables

Table S1. CDC RT-qPCR panel: primers and probes

Name	Description	Oligonucleotide sequence (5'→3')	Label	Final conc.
2019-nCoV_N1-F	2019-nCoV_N1 Forward Primer	5’-GAC CCC AAA ATC AGC GAA AT-3’	None	500nM
2019-nCoV_N1-R	2019-nCoV_N1 Reverse Primer	5’-TCT GGT TAC TGC CAG TTG AAT CTG-3’	None	500nM
2019-nCoV_N1-P	2019-nCoV_N1 Probe	5’-FAM-ACC CCG CAT TAC GTT TGG TGG ACC-BHQ1-3’	FAM, BHQ-1	125nM
2019-nCoV_N2-F	2019-nCoV_N2 Forward Primer	5’-TTA CAA ACA TTG GCC GCA AA-3’	None	500nM
2019-nCoV_N2-R	2019-nCoV_N2 Reverse Primer	5’-GCG CGA CAT TCC GAA GAA-3’	None	500nM
2019-nCoV_N2-P	2019-nCoV_N2 Probe	5’-FAM-ACA ATT TGC CCC CAG CGC TTC AG-BHQ1-3’	FAM, BHQ-1	125nM
RP-F	RNAse P Forward Primer	AGA TTT GGA CCT GCG AGC G	None	500nM
RP-R	RNAse P Reverse Primer	GAG CGG CTG TCT CCA CAA GT	None	500nM
RP-P	RNAse P probe	FAM – TTC TGA CCT GAA GGC TCT GCG CG – BHQ-1	FAM, BHQ-1	125nM

Table S2. Clinical sample characterization by CDC RT- qPCR, based on N1 CDC assay

**Categories**[a]	**RT-qPCR C_t range N1 assay (cycles)**	**RNA concentration (copies/reaction)**[b]	**RNA concentration (copies/mL of Lysis Buffer)**[c]	**Samples per category (*n*)**
High	14 – 20.9	$1 \times 10^7 - 1 \times 10^5$	$2 \times 10^9 - 1 \times 10^7$	48
Upper-medium	21 – 24.9	$1 \times 10^5 - 8 \times 10^3$	$1 \times 10^7 - 1 \times 10^6$	40
Lower-medium	25 – 30.9	$8 \times 10^3 - 1 \times 10^2$	$1 \times 10^6 - 1 \times 10^4$	38
Low	31 – 37.6	$1 \times 10^2 - 1 \times 10^0$	$1 \times 10^4 - 1 \times 10^2$	35
Negative	Not detected	–	–	245
Total	-			406

[a]All samples tested positive by the RNAse P assay. [b]Estimated concentration (copies/reaction) based on RT-qPCR standard curve $y = -3.30\ln(x) + 37.98$. [c]Estimated concentration (copies/mL of Lysis Buffer) considering 140µL of sample input and 80µL eluted volume.

Supplementary figures

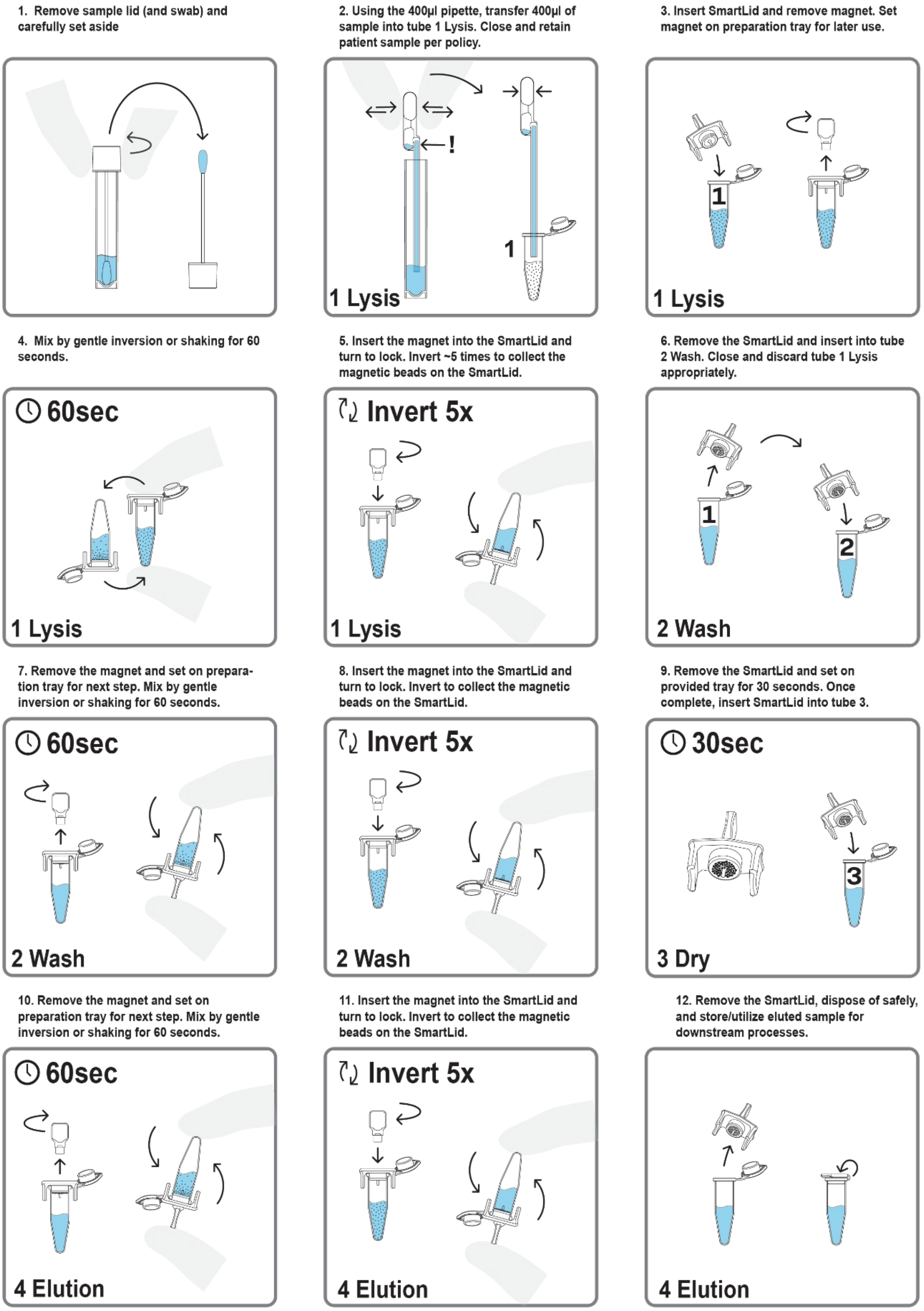

Figure S1. SmartLid workflow

Schematic representation of sample workflow from swabbed sample to eluted product using SmartLid technology.

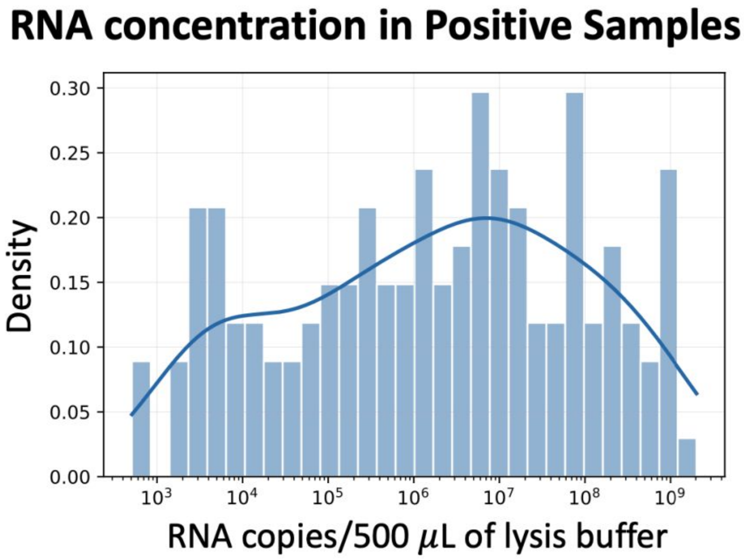

Figure S2. RNA concentration in clinical samples

Estimated SARS-CoV-2 RNA concentration (copies/500 µL of storage buffer COPAN eNAT containing VTM) across positive samples.
